# The Effect of Stabilized Rice Bran Addition on Physicochemical, Sensory, and Techno-Functional Properties of Bread

**DOI:** 10.3390/foods11213328

**Published:** 2022-10-23

**Authors:** Cindy Espinales, Adriana Cuesta, Javier Tapia, Sócrates Palacios-Ponce, Elena Peñas, Cristina Martínez-Villaluenga, Alexander Espinoza, Patricio J. Cáceres

**Affiliations:** 1 Facultad de Ingeniería en Mecánica y Ciencias de la Producción, Escuela Superior Politécnica del Litoral, ESPOL, Campus Gustavo Galindo, Km. 30.5 Vía Perimetral, Guayaquil 090902, Ecuador; 2 Department of Food Technology and Biotechnology, Institute of Food Science, Technology and Nutrition (ICTAN-CSIC), José Antonio Novais 10, 28040 Madrid, Spain; 3 Facultad de Ingeniería Química, Universidad de Guayaquil, Guayaquil 090510, Ecuador

**Keywords:** functional foods, *Oryza sativa*, rice bran, bread, bioactive compounds

## Abstract

Rice bran (RB) is a valuable byproduct derived from rice milling that represents an excellent opportunity for dietary inclusion. Bioactive components with antioxidant potential have been reported in RB, gaining the considerable attention of researchers. However, RB requires a stabilization process after milling to prevent it from becoming rancid and promote its commercial consumption. The aim of this study was to evaluate the effects of substituting stabilized rice bran (SRB) for wheat flour at levels of 10, 15, 20 and 25% on the proximate composition, dietary fiber, dough rheology, antioxidant properties, content of bioactive compounds, and sensory attributes of white wheat-based bread. Results indicated that the incorporation of SRB increased the bread’s insoluble dietary fiber, phytic acid, total polyphenol content, γ-oryzanol, γ-aminobutyric acid, and antioxidant properties, while decreased its water absorption capacity, elasticity, volume, β-glucans, and soluble dietary fiber content. Moreover, substituting wheat flour for SRB at levels higher than 15% affected sensory attributes, such as color, odor, flavor, and softness. This study highlights the potential application of SRB flour in bread-making to increase nutritional, and functional properties of white wheat bread.

## 1. Introduction

Rice (*Oryza sativa* L.) is an important staple food worldwide that is mostly consumed in its refined form, annually generating millions of tons of by-products such as rice bran (RB) [1], the layer between the rice kernel and husk. RB is an excellent source of carbohydrates (34–52%), lipids (15–22%), protein (10–16%) and fiber (7–11%) [2]. Moreover, RB contains high levels of bioactive compounds including γ-oryzanol, phytosterols and polyphenols [3,4]. The consumption of RB has been demonstrated to be associated with the prevention and control of chronic diseases such as diabetes and cardiovascular disorders [5]. Furthermore, RB exhibits antioxidant, anti-inflammatory, and anti-cancer activity [6,7]. Despite its high nutritional value and vast health-promoting properties, RB is mostly used for animal feeding, and its utilization for human consumption is very limited because RB consumption can be associated with the high content of phytic acid, which is related with mineral deficiency in humans due to its chelating effect [8]. However, studies have shown that this compound has favorable effects against different types of cancer, diabetes, atherosclerosis, and coronary diseases [9]. Therefore, the revalorization of RB as an ingredient to increase the nutritional value and healthy potential of foodstuffs is of undoubted interest. However, due to its high lipid content and lipase activity, RB is prone to oxidation leading to undesired properties during storage (rancidity and unpleasant taste) [10], and it should be thermally stabilized before its incorporation into food products. Thermal treatment is the most common method of stabilizing RB due to the high temperature used to effectively denature lipases [11]. Moreover, autoclaving, which is the combination of high temperature and high pressure, has been demonstrated to be the most effective way to inactivate RB lipase with no significant influence on the bioactive compounds such as γ-oryzanol [12].

Recent studies have shown that stabilized RB (SRB) improves the nutritional value and health-promoting properties of different baked foodstuffs such as bread [13,14,15], biscuits [14,16] and muffins [17]. However, most of these studies have shown that levels of replacement of wheat flour with more than 10% SRB adversely affects the sensory and texture properties of food products [13].

Bread is one of the oldest and most consumed foodstuffs worldwide and is usually made from wheat flour, salt, and water with or without leavening agent. White bread provides carbohydrates and energy, but it lacks some essential amino acids, micronutrients, fiber and bioactive compounds [18,19]. Presently, most of the efforts of the food manufacturing industry and food researchers are directed towards fulfilling the needs and expectations of consumers [20] who increasingly demand foods that improve their nutritional status, health and well-being [21]. Replacing refined wheat flour with other cereal-based and nutrient-rich ingredients during bread elaboration offers a promising approach to enhance the nutritional quality of bread while providing a healthier product and a source of innovation for the food industry. SRB represents an interesting raw material to achieve these purposes. However, Irakli et al. [22] revealed that substitution of wheat flour by SRB at levels higher than 10% negatively affected the crumb color and texture of the bread. Similarly, it was observed that incorporation of SRB at levels beyond 15% caused a darker color of bread crust and redness (a+) of biscuits and had a negative effect on sensory and texture properties of both foods [14]. Therefore, the aim of the present study was to incorporate SRB at different concentrations (10%, 15%, 20% and 25%) during breadmaking to obtain bread formulations with good physicochemical, sensory and techno-functional properties, thus overcoming the limitations of previous studies regarding the poor sensory quality of breads when high SRB concentrations were used. The novelty of this work lies in the evaluation, for the first time, of a wide range of bioactive compounds, together with nutritional and physico-chemical properties in SRB-added breads.

## 2. Materials and Methods

### 2.1. Chemical, Reagents and Standards

All chemicals used were of analytical grade and were provided by Sigma-Aldrich (Madrid, Spain) unless otherwise specified. Total Dietary Fiber (K-TDFR) and β-Glucan (K-BGLU) Assay Kits were purchased from Megazyme (Wicklow, Ireland).

### 2.2. Stabilization of RB

Milled RB was provided by a local cereal processing association (Montubios Rio Cu-lebra Association, Cascajal, Ecuador), and it was immediately stored in jute bags at 18 °C to avoid oxidation for further use. RB stabilization was carried out in an autoclave (ARIS-TEK 16L, México City, Mexico) at 120 °C and 5 psi for 20 min. After removal of lumps formed during the heat treatment, SRB was air-dried at 70 °C for 20 min. Finally, to facilitate the mixing process and to avoid lumps, SRB was sieved using a 500 µm screen (35-mesh) and it was packed in polyethylene bags and stored at ambient temperature (28 ± 2 °C).

### 2.3. Manufacture of Bread Supplemented with SRB

#### 2.3.1. Ingredients

Commercial refined wheat flour (Superior, Guayaquil, Ecuador), canola oil (La Fabril, Manta, Ecuador), refined cane sugar (Valdez, Milagro), refined salt (Ecuasal, Guayaquil, Ecuador), dry yeast *(Saccharomyces cerevisiae*) (Fleischmann, Durán, Ecuador), and calcium propionate (CriFood, Guayaquil, Ecuador) were purchased from a local supermarket in Guayaquil (Ecuador).

#### 2.3.2. Breadmaking Process

Two kg of refined wheat flour was mixed with sugar (120 g), salt (40 g), calcium propionate (14 g) and SRB flour, which substituted wheat flours at different proportions (10, 15, 20 and 25% related to wheat flour weight) (Table 1). Commercial white wheat bread formulated with 100% refined wheat flour was used as control. The solid ingredients were sieved through a 10-mesh sieve, mixed manually for 10 min and packaged in polyethylene bags, and all bags were labeled according to their composition. The different pre-mix formulations were stored in a cool place for further use.

**Figure 1:**
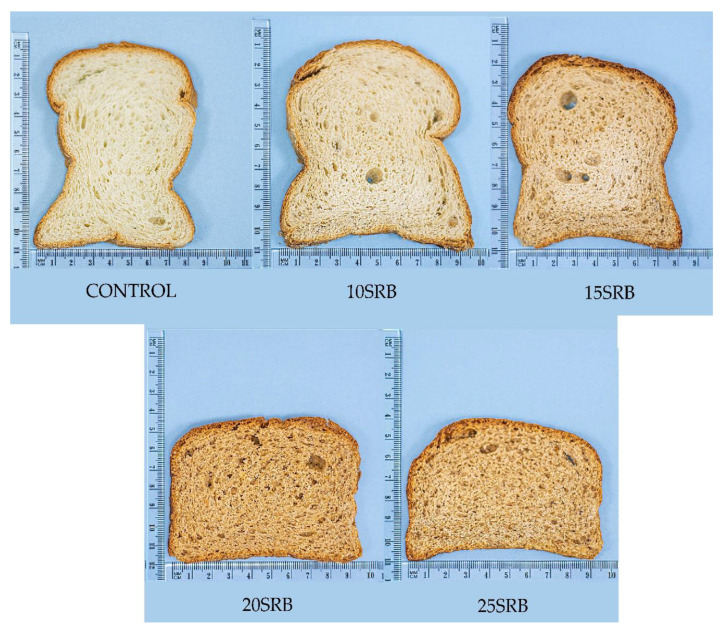
Breads formulated with different percentages of SRB. Abbreviations: 10SRB: formulation with 10% of SRB; 15SRB: formulation with 15% of SRB; 20SRB: formulation with 20% of SRB; 25SRB: formulation with 25% of SRB. The scale of the figure is in cm.

White wheat breads (tin loaf type) were produced by applying a direct method, combining the pre-mix formulations with wet ingredients and yeast (Table 1) using a kitchen mixer (Xingfeng^®^ B10GF, Jiangmen, China) at 566 rpm for 10 min. Doughs were left to stand for 10 min, and then doughs were divided into 500 g pieces before they were manually rounded and loaded into baking tins. Doughs were fermented for 90 min at room temperature, and then they were baked at 185 °C for 30 min in a gas oven (Fritega S.A., CP-COG, Guayaquil, Ecuador). All breads were baked in triplicate using the same oven. Breads (Figure 1) were cooled at room temperature for 60 min and immediately they were packaged in polyethylene bags and stored at room temperature for 22 h for further analysis. An amount of 20 g of each bread was freeze-dried (FreeZone 6 Liter, Labconco, Kansas City, MO, USA) for further nutritional and bioactive compound analysis.

### 2.4. Characterization of Doughs

#### 2.4.1. Dough Mixing Properties

The rheological properties of doughs during mixing were evaluated using a farinograph (Mixolab 2, Chopin Technologies, Villeneuve-la-Garenne, France). The measurements were performed following the manufacturer’s protocol (mixing speed: 80 rpm; dough weight: 75 g; tank temperature: 30 °C; dough temperature: 30 °C; analysis time: 30 min). To evaluate consistency, the doughs were subjected to a double-kneading pressure at increasing temperature. The parameters analyzed were water absorption capacity, development time, stability and weakening.

#### 2.4.2. Dough Extension Properties

The physical properties of doughs during extension were determined in an alveograph (Alveolab, Chopin Technologies, Villeneuve-la-Garenne, France). The analysis was performed according to the hydration constant method (ISO 27971: 2015) and allowed to evaluate the ability of doughs to tolerate stretching and to hold the CO_2_ produced without collapsing during mixing. The following parameters were analyzed: dough tenacity (P), elasticity (L), and deformation energy (W).

#### 2.4.3. Dough pH

The pH values of doughs were determined after fermentation according to AOAC (945.42 method). All pH values were determined in triplicate.

### 2.5. Characterization of breads

#### 2.5.1. Physical Characteristics

Breads weights (g) were determined on an analytical balance (BAS 31 plus, BOECO, Hamburg, Germany), and central high was measured using a vernier caliper (Kingcompany, Spurtar, Beaverton, OR, USA). Bread volume (cm^3^) was quantified by the measurement of the volume by rapeseed displacement (AACC 10.05.01 method).

#### 2.5.2. Proximate Composition

Moisture, protein, fat, crude fiber and ash were determined according to AOAC methods 926.05, 950.36, 935.38, 978.10 and 930.22, respectively. Available carbohydrates were estimated by difference: 100 − (proteins (g/100 g d.m.) + fat (g/100 g d.m.) + water (g/100 g) + dietary fiber (g/100 g d.m.) + ash (g/100 g d.m.) [23].

#### 2.5.3. Dietary Fiber

Two grams of dried bread samples were defatted with a Soxhlet extractor using petroleum benzine as the extracting solvent. The analyses of soluble, insoluble, and total dietary fiber were carried out in triplicate according to the official enzymatic–gravimetric AACC 32-07.01 method, using a commercial kit (TDFR-200A, Megazyme, Wicklow, Ireland).

#### 2.5.4. Phitic Acid Content

Phytic acid content was quantified as described by Cornejo et al. [24], with slight modifications. Briefly, 50 mg of dried samples were placed in a vial and mixed with 1 mL of 1 M HCl. The vial was covered with aluminum foil and was heated at 92 °C for 45 min in a glycerol bath under constant agitation (10× *g*). Then, vials were cooled in an ice bath for 10 min and centrifuged at 13,000× *g* for 5 min. Then 0.25 mL of the supernatant were collected and mixed by stirring with 1 mL of ultrapure water. An aliquot of 0.4 mL of sample, standard (phytic acid solution in 0.2 M HCl) or blank (0.2 M HCl) were added to a vial with 0.8 mL of ferric solution (0.025 g of FeCl_3_ in 250 mL of 0.2 M HCl). The mixture was heated at 92 °C for 45 min in a glycerol bath under constant agitation (10× *g*), and then, it was cooled down in an ice bath for 15 min. After centrifugation at 13,000× *g* at room temperature for 5 min, an aliquot of the supernatant (0.6 mL) was added to 0.8 mL of the complexing reagent (0.5 g of 2,20-bipyridine and 65 µL of thioglycolic acid dissolved in 50 mL of 0.2 M HCl), and absorbance was read at 540 nm using a microplate reader (BioTek Instruments, Winooski, VT, USA) controlled by the Gene 5TM software version 1.1 (BioTek Instruments).

#### 2.5.5. β-Glucan Content

The content of β-glucan was quantified according to the AOAC method 995.16 using a commercial enzyme kit (K-BGLU 01/21, Megazyme, Wicklow, Ireland).

#### 2.5.6. γ-Oryzanol Content

γ-oryzanol was quantified as described by Srisaipet and Nuddagul [25] with slight modifications. Briefly, 1 g of dried samples was diluted with 4 mL of isopropanol and the extraction was performed in agitation (200 rpm) at room temperature overnight. After centrifugation (3000 rpm, 5 min, room temperature), supernatants were collected, and the absorption spectra of γ-oryzanol was measured using a microplate reader (BioTek Instruments, Winooski, VT, USA) controlled by the Gene 5TM software version 1.1 (BioTek Instruments). A standard curve (0–50 mg/L) was prepared from a 0.1 mg/mL γ-oryzanol solution.

#### 2.5.7. Total Soluble Phenolic Content

Total content of free phenolic compounds was measured in bread methanolic ex-tracts by the Folin–Ciocalteu’s reagent at 739 nm as previously reported [26].

#### 2.5.8. γ-Aminobutyric Acid (GABA)

GABA was determined in water extracts obtained from freeze-dried bread samples as previously reported [26]. Briefly, an aliquot (50 μg) of extract was combined with an allyl-L-glycine solution (10 μL, internal standard) and derivatized with phenyl isothiocyanate (30 μL). Then, the resultant extract was dried in a vacuum concentrator, and the sample was solubilized in 0.1 M ammonium acetate, pH 6.5 for GABA quantification, which was performed using high-performance liquid chromatography (HPLC) coupled to a diode array detector [26]. Analyses were carried out in duplicate.

#### 2.5.9. Oxygen Radical Absorbance Capacity (ORAC)

Radical scavenging activity was quantified by fluorescence in methanolic extracts according to the oxygen radical absorbance capacity (ORAC) assay [26]. Fluorescence was determined in a microplate reader (BioTek, Winooski, VT, USA) at emission and excitation wavelengths of 520 nm and 485 nm, respectively. Trolox was used as standard (concentration range: 0–160 µM).

#### 2.5.10. Sensory Evaluation

The sensory properties of breads were evaluated using a descriptive analysis by 5 trained panelists (2 females and 3 males, aged 33 to 47). Sensory evaluation was carried out on the same day of baking. The different bread formulations were served to panelists on white plastic plates at room temperature. Breads evaluations were performed by each judge individually in isolated booths. Water was provided to rinse the mouth between evaluations. Color, odor, flavor softness, overall acceptability and appearance were the attributes analyzed on a 5-point hedonic scale (2 = like very much, 1 = like moderately, 0 = indifferent, −1 = dislike moderately, −2 = dislike very much).

### 2.6. Statistical Analysis

The results of all analyses are the average of at least triplicate measurements of three experimental replicates. Data were subjected to analysis of variance (ANOVA) using Statgraphics 19 software (Statistical Graphics Corp., Rockville, MD, USA). Tukey’s multiple-range test was used to determine significant differences between means at a confidence level of 95% (α = 0.05) in analyzing sensory evaluation results.

## 3. Results and Discussion

### 3.1. Dough Mixing Properties

The mixing properties of dough formulations as a result of the addition of SRB at different levels are shown in Table 2. The control dough was characterized by a high-water absorption capacity (66.4%), typical of breads made from 100% refined wheat flour. The water absorption capacity was significantly reduced (*p* < 0.05) as the SRB ratio in the dough increased, in agreement with results previously observed by other authors in breads enriched with RB [14,27,28]. Water absorption plays a key role in the development of the gluten network and subsequently in bread quality [29]. The observed variation in water absorption capacity after SRB addition may be explained by the substitution of gluten containing wheat flour with SRB that limited water–fiber interaction, making the dough more hydrophobic [14]. Moreover, the high fat content of SRB could contribute to the lower water absorption capacity observed in SRB-enriched breads, as previously observed [28].

SRB substitution also reduced development time, that is, the time elapsed from the addition of water to point of maximum consistency before the beginning of weakening [14]. The dough development time is closely linked to the gluten characteristics of wheat flour, since gliadins and glutenin provide the dough its characteristics elasticity and resistance and allow the formation of a protein network that other types of flour do not exhibit [30,31]. The results obtained clearly show that the addition of SRB flour interrupts the starch–protein matrix, which can decrease dough elasticity and cause a weakening of the dough during mixing, results in close agreement with results found by other authors after incorporating SRB [28] and other gluten-free matrices such as pea flour [29]. The decreased development time was accompanied by increased stability time, suggesting that the shorter the dough’s development time, the higher the stability of the dough.

### 3.2. Dough Extension Properties

The results obtained from the alveograph analysis are shown in Table 3. Tenacity, elasticity and deformation energy of doughs decreased as the SRB replacement ratios in bread increased. The reduction of gluten content as a result of SRB incorporation causes a lower dough strength and smaller capacity to resist deformation, results also observed in other studies when wheat flour was replaced with SRB [32,33]. The reduced deformation energy observed for doughs containing SRB was attributed to the lower glutenin content and dough strengths as previously reported in breads enriched in maize gluten feed, the by-product of the wet milling of maize grain [34] and in breads formulated with amaranth and finger millet [35].

### 3.3. Doughs pH

The pH of the doughs ranged between 5.43 and 5.57 (Table 4). The addition of SRB to bread formulation did not influence the pH of doughs after fermentation. Similar pH values were observed in RB sourdoughs fermented with yeast or yeast combined with lactic acid bacteria [36]. According to Larsson [37], the loaf volume of baked bread increases at pH 4.65–6.15 due to the effect of dough fermentation and decreases at higher pH. The values obtained were also similar to the standard pH (5.95–5.98) of industrial white wheat bread with the dough-resting time of 30 min [38].

### 3.4. Physical Properties of Bread

Notable differences regarding bread volume and height were observed between the different SRB bread formulations (Table 5). The volume of bread ranged from 1061 cm^3^ in control bread to 750 cm^3^ in breads with the highest SRB level (25%). Reductions in bread volume between 7% and 29% were observed depending on the percentage of SRB incorporation. The characteristic high volume of wheat-based breads can be attributed to their gluten content, which allows the retention of the gas produced during fermentation and reflects the ability of bread dough to expand [39]. The replacement of wheat flour with SRB flour causes a gluten-dilution effect and the disruption of the gluten network by the reaction of fiber with gluten proteins [35], thus reducing the bread’s air-holding capacity and, consequently, the bread volume. Similarly, the height of bread loaves decreased as the SRB amount increased, from 3% in 10SRB bread to 28% in 25SRB formulation, due to the lower gluten content compared with the control, corroborating the results observed for bread volume.

### 3.5. Nutritional Composition

The nutritional compositions of the control and experimental breads with different levels of SRB are shown in Table 6. According to Schopf and Scherf [40], moisture content is an important parameter in bread quality. Experimental breads containing SRB had significantly higher moisture (33.7–33.9 g/100 g) than the control one (32.5 g/100 g), except for 10SRB with significant lower moisture (*p* ≤ 0.05). Our results regarding moisture content in breads containing SRB are similar to those reported in breads fortified at 30% with wheat, sorghum, maize, and rice [39,40].

SRB addition to bread formulation did not influence the protein content of bread (*p* > 0.05). This result contrasts with previous studies reporting a small decrease in the protein content in breads with cereal bran fortification up to 30% attributed to heat treatment during bread production [39,40]. These differences could be explained by different rates of bran supplementation and the protein content in the raw materials. Rice brans contain lower protein levels compared with oat (17.63 g/100 g), wheat (13.2 g/100 g), sorghum (7.01 g/100 g) and maize (6.79 g/100 g) [39,41,42]. Fat content increased gradually with increasing levels of SRB (from 6.64% in control bread to 9.78% in bread 25SRB), due to the high fat content of SRB accounting for 20% of total weight [13,43]. Carbohydrates were decreased with increasing levels of SRB, due to the dilution of starch content in bread caused by addition of SRB mainly composed by fiber (13.9–24.4%), fat (18–25.5%), protein (12.7–16.3%) and ash (10–11.89%) [44]. On the other hand, ash content increased gradually as function of SRB supplementation level in bread formulation, attributed to the mineral enrichment of bread caused by SRB addition [41].

Phytic acid, mainly located in the bran layer of rice, has a strong ability to bind to metal ions, such as zinc, calcium, magnesium and iron [45], forming insoluble complexes in the digestive tract that negatively affect the mineral bioavailability [44,45]. Nonetheless, endogenous phytase activity of wheat contributes to the gradual reduction of phytic acid as the bread-making process advances [46]. During the bread-making process, the extent of phytic acid enzymatic degradation depends on dough pH, fermentation temperature and time and baking time being not generally enough to extensively improve the bioavailability of minerals [47]. In the present study, phytic acid content increased proportionally to the SRB percentage added to the bread formulation (Table 6). Control bread showed the lowest value (0.18 g/100 g d.m.), while experimental bread 25SRB had the highest phytic acid content (1.22 g/100 g d.m.). Compared with cereal-bran enriched breads reported in the literature, the phytic acid content in 10SRB was within the reported range (0.4–0.6 mg/100 g) [39], while higher SRB substitutions (15–25%) led to breads with remarkably higher phytic acid levels (0.86–1.22 g/100 g).

Total dietary fiber (TDF) increased gradually with increasing amounts of SRB in the bread formulation compared with control bread (Table 6, *p* ≤ 0.05). The distribution of insoluble (IDF) and soluble (SDF) dietary fiber changed as a consequence of SRB supplementation. For instance, 95% of TDF was insoluble in bread 25SRB, while control bread was mainly composed by SDF accounting for 63% of the content of TDF. IDF increased proportionally with the level of SRB substitution in bread (Table 6, *p* ≤ 0.05) whereas an opposite effect was observed for SDF. This effect is attributed to the chemical composition of SRB composed of 20–30% of TDF, of which nearly 90% represents the insoluble fraction [48]. Additionally, Tuncel et al. [13] reported low SDF:IDF ratio for breads with SRB substitution.

β-glucans are part of cereals’ dietary fiber, and they are found as (1–3)-(1–4)-β-glucans. These compounds provide nutritional benefits such as controlling postprandial blood pressure and insulin resistance [49]. The content of β-glucans in bread with partial SRB substitution is presented in Table 6. β-glucans content decreased proportionally with increasing substitution levels of SRB in bread, except for 10SRB bread, which had similar β-glucan content (1.70%) to in of control bread (1.69%). According to Jung et al. [49], β-glucans in rice bran varies from 0.18 to 0.57%, a lower concentration range than the β-glucan content of wheat flour (~1%) [48,49]. Therefore, the β-glucan content reduction observed in SRB bread fortification could be explained by a dilution effect [50].

### 3.6. Bioactive Compounds and Antioxidant Activity

**Figure 2:**
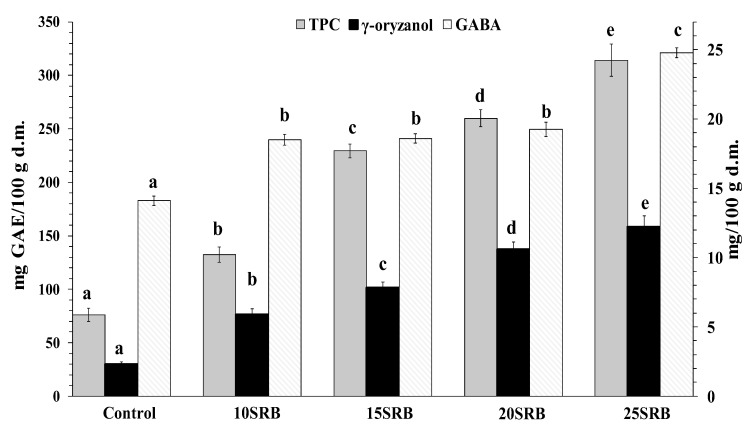
Total phenolic compounds (mg GAE/100 g d.m.), γ-oryzanol (mg/100 g) and GABA (mg/100 g) content in white wheat bread with stabilized rice bran (SRB) (n = 3). Abbreviations: 10SRB, formulation with 10% of SRB; 15SRB, formulation with 15% of SRB; 20SRB, formulation with 20% of SRB; 25SRB, formulation with 25% of SRB. The different lowercase letters indicate significant difference among mean values within a same compound (*p* ≤ 0.05 according to Duncan’s test).

**Figure 3:**
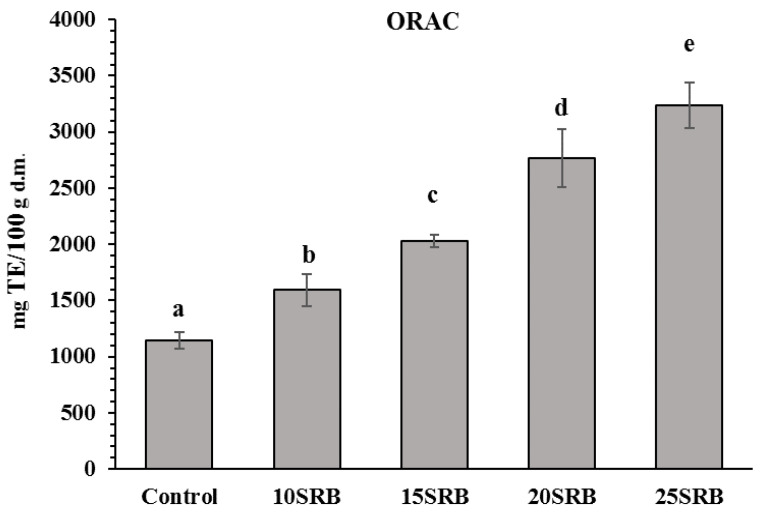
Antioxidant activity in white wheat bread with stabilized rice bran (SRB) (n = 3). Abbreviations: 10SRB, formulation with 10% of SRB; 15SRB, formulation with 15% of SRB; 20SRB, formulation with 20% of SRB; 25SRB, formulation with 25% of SRB. The different lowercase letters indicate significant difference among mean values (*p* ≤ 0.05 according to Duncan’s test).

SRB bread fortification significantly affected the amount of bioactive compounds (Figure 2) and antioxidant activity (Figure 3).

γ-Oryzanol, the strongest antioxidant in rice bran, is a mixture of ferulic acid esters of triterpene alcohols and sterols. Major physiological effects have been associated with γ-oryzanol intake including hypocholesterolemic activity [51]. The results presented in Figure 2 indicate that SRB bread fortification significantly enhanced γ-oryzanol content, reaching amounts that oscillated from 6.0 to 12.2 mg/100 g. This enrichment is attributed to the high abundance of γ-oryzanol in SRB that can oscillate from 118.80 to 311.07 mg/100 g depending on the rice variety [49]. The methods used for rice bran fractionation and subsequent stabilization are additional factors that may introduce variations in the γ-oryzanol content of rice bran [52]. In addition, bread baking also negatively affects γ-oryzanol content in breads due to its thermal degradation [24].

Polyphenols are present in food products derived from plant sources. Their consumption is associated with a low risk of chronic diseases such as cardiovascular disease, aging, cancer and Alzheimer [49,52,53,54]. As can be seen in Figure 2**,** the TPC of breads containing SRB increases significantly and in a dose-dependent manner with the concentration of added SRB (*p* < 0.05). These findings are consistent with a previous study performed by Irakli et al. [22], who concluded that the inclusion of SRB in wheat bread enhanced free and bound phenolic compounds in bread. The TPC of SRB fortified breads obtained in the present study fall in the range of reported amounts for breads made from wheat flour–wheat bran blends at a ratio between 10–30% of bran (110.6–258.9 mg/100 g for bread crumb and 298.8–487.4 mg GAE/100 g for bread crust) [1].

Correlation analysis showed a strong positive correlation between γ-oryzanol and total phenolic content (Pearson’s correlation coefficient = 0.95). However, in the study of Jung et al. [49], the correlation obtained between γ-oryzanol and the total phenol content of RB was low (r = 0.2396), suggesting that a better correlation could be obtained with the stabilization of rice bran.

GABA content variation as a function of SRB substitution ratio is shown in Figure 2. SRB-fortified breads showed higher amounts of GABA (14.11 mg/100 g d.m.) compared with control bread (24.79 mg/100 g d.m.). The highest GABA content was observed for the highest substitution ratio (25SRB bread). Wheat flour has a low GABA content whose concentration varies from 3 to 78 mg/100 g depending on wheat variety [55]. A previous study has demonstrated that RB is a source (634 mg/100 g) of GABA [56]. Taken together, increased GABA content in bread may be mainly attributed to SRB fortification.

The ORAC results for the SRB fortified breads and control is shown in Figure 3. There was a significant variation in ORAC from 165 to 325 mg TE/100 g d.m. in SRB-fortified breads. ORAC in breads increased proportionally with increasing rates of SRB substitution. The results are consistent with those presented in the study by Irakli et al. [22], who observed that the antioxidant capacity of the samples with rice bran flour substitution from 10 to 25 % ranged from 100 to 350 mg TE/100 g d.m. Furthermore, Aktaş and Akın [57] also reported that the antioxidant activity found in rice bran was higher compared with wheat flour and corn bran incorporated in fermented cereal-based food products.

### 3.7. Sensory Analysis

**Figure 4:**
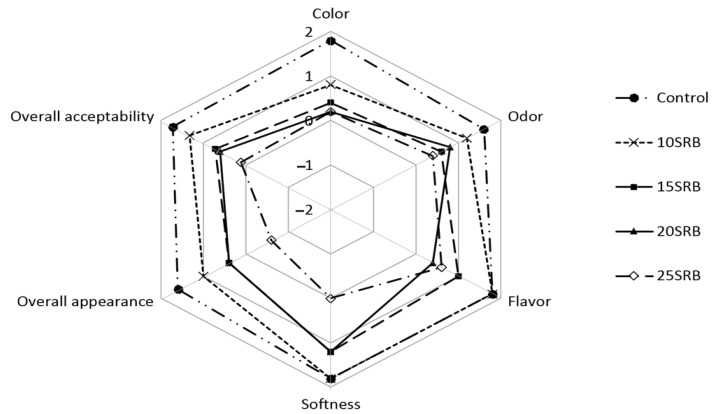
Sensory analysis of white wheat bread formulated with different levels of stabilized rice bran (SRB). Abbreviations: 10SRB, formulation with 10% of SRB; 15SRB, formulation with 15% of SRB; 20SRB, formulation with 20% of SRB; 25SRB, formulation with 25% of SRB.

Sensory attributes of bread substituted with different percentages of SRB flour are shown in Figure 4. The sensory properties of breads were significantly (*p* < 0.05) affected by the incorporation of SBR in comparison to control bread, and the magnitude of the change depended on the SRB concentration used. Formulation 10SRB exhibited sensory properties close to those of control bread, especially flavor and softness, in agreement with previous studies [58]. However, the addition of higher SRB concentrations (15–25%) had a negative impact on all sensory parameters of breads, particularly on color, odor and flavor, with these breads showing significantly (*p* < 0.05) lower scores for overall acceptability than control bread. The inclusion of SRB increases the fiber content but decreases gluten proteins, resulting in lower loaf volumes and harder crumbs, which could explain the negative impact of SRB on texture and bread quality [58,59,60]. These challenges in the utilization of SRB in breadmaking have been already overcome by SRB–wheat flour sourdough fermentation, which improved consumer acceptability based on higher loaf volumes, softer crumbs, a more cohesive and moister mouthfeel and reduced beany and cooked rice flavors [61].

## 4. Conclusions

The replacement of wheat flour with SRB in bread significantly increased total antioxidant activity, total dietary fiber, ashes and bioactive compounds such as GABA and γ-oryzanol. However, the quality of wheat bread was greatly influenced by the amount of added SRB. The higher amount of added SRB gave lower product quality, which resulted in reduced viscoelastic properties, specific volume and height of bread. The results indicated that substituting wheat flour up to 15% affects the overall dough physical properties and bread sensory attributes. In this sense, more studies about the possible interfering effects of rice bran composition on dough rheology are required.

## Figures and Tables

**Table 1:** Formulations for control and SRB-enriched breads (wheat flour + SRB = 100%).

Ingredients (On Flour + SRB%)	Bread Formulations				
	Control	10SRB	15SRB	20SRB	25SRB
Wheat flour	100	90	85	80	75
SRB	0	10	15	20	25
Sugar	6	6	6	6	6
Canola oil	8	8	8	8	8
Yeast	4	4	4	4	4
Water	60	57.3	55.9	55	53.5
Salt	2	2	2	2	2
Calcium propionate	0.7	0.7	0.7	0.7	0.7

Abbreviations: SRB, stabilized rice bran; 10SRB: formulation with 10%; 15SRB: formulation with 15%; 20SRB: formulation with 20%; 25SRB: formulation with 25% of wheat flour substituted by SRB.

**Table 2:** Mixing properties of control and experimental doughs formulated with different levels of SRB substitution.

Dough Formulation	Water Absorption (g/100 g)	Development Time (min)	Stability (min)	Weakening (mN·m)
Control	66.40 ± 0.33 ^e^	3.77 ± 0.12 ^b^	24.12 ± 0.85 ^a^	49.00 ± 6.55 ^b^
10SRB	63.60 ± 0.42 ^d^	3.63 ± 0.06 ^ab^	28.15 ± 0.15 ^b^	68.67 ± 1.53 ^c^
15SRB	62.25 ± 0.29 ^c^	3.48 ± 0.03 ^a^	29.45 ± 0.38 ^c^	65.01 ± 2.00 ^c^
20SRB	61.25 ± 0.29 ^b^	3.98 ± 0.08 ^c^	35.10 ± 1.15 ^d^	45.00 ± 1.73 ^ab^
25SRB	59.80 ± 0.24 ^a^	3.57 ± 0.10 ^a^	37.02 ± 0.23 ^e^	40.33 ± 2.52 ^a^

Data are the mean ± standard deviation. Different superscript letters within a column indicate significant differences (*p* ≤ 0.05). Abbreviations: SRB, stabilized rice bran; 10SRB: formulation with 10% of SRB; 15SRB: formulation with 15% of SRB; 20SRB: formulation with 20% of SRB; 25SRB: formulation with 25% of SRB.

**Table 3:** Extension properties of control and experimental doughs formulated with different levels of SRB substitution.

Dough Formulation	Tenacity (mm H_2_O)	Elasticity (mm)	Deformation Energy (× 10^−4^ J)
Control	184.33 ± 5.51 ^d^	48.17 ± 1.04 ^b^	375.70 ± 5.13 ^d^
10SRB	170.33 ± 3.51 ^c^	37.16 ± 3.01 ^a^	254.82 ± 5.01 ^c^
15SRB	158.00 ± 3.04 ^b^	33.50 ± 3.04 ^a^	213.17 ± 3.25 ^b^
20SRB	159.67 ± 5.77 ^b^	37.35 ± 2.52 ^a^	207.67 ± 4.16 ^b^
25SRB	148.50 ± 4.09 ^a^	34.70 ± 3.06 ^a^	195.66 ± 2.75 ^a^

Data are the mean ± standard deviation. Different superscript letters within a column indicate significant differences (*p* ≤ 0.05). Abbreviations: SRB, stabilized rice bran; 10SRB: formulation with 10% of SRB; 15SRB: formulation with 15% of SRB; 20SRB: formulation with 20% of SRB; 25SRB: formulation with 25% of SRB.

**Table 4:** pH values of control and experimental doughs formulated with different levels of SRB substitution.

Dough Formulation	pH
Control	5.43 ± 0.04 ^a^
10SRB	5.53 ± 0.02 ^a^
15SRB	5.45 ± 0.06 ^a^
20SRB	5.51 ± 0.02 ^a^
25SRB	5.57 ± 0.03 ^a^

Data are the mean ± standard deviation. Same superscript letters within a column indicate no significant differences (*p* ≤ 0.05). Abbreviations: SRB, stabilized rice bran; 10SRB: formulation with 10% of SRB; 15SRB: formulation with 15% of SRB; 20SRB: formulation with 20% of SRB; 25SRB: formulation with 25% of SRB.

**Table 5:** Physical characteristics of control and experimental breads formulated with different levels of SRB substitution.

Bread Formulation	Volume (cm^3^)	Center Height (cm)
Control	1061.33 ± 10.26 ^e^	10.21 ± 0.11 ^d^
10SRB	985.36 ± 14.19 ^d^	9.90 ± 0.10 ^d^
15SRB	900.10 ± 20.00 ^c^	8.41 ± 0.10 ^c^
20SRB	863.36 ± 6.09 ^b^	7.96 ± 0.04 ^b^
25SRB	750.20 ± 9.70 ^a^	7.30 ± 0.36 ^a^

Data are the mean ± standard deviation. Different superscript letters within a column indicate significant differences (*p* ≤ 0.05). Abbreviations: SRB, stabilized rice bran; 10SRB, formulation with 10% of SRB; 15SRB, formulation with 15% of SRB; 20SRB, formulation with 20% of SRB; 25SRB, formulation with 25% of SRB.

**Table 6:** Nutritional composition (expressed in g/100 g d.m.) of control and experimental breads formulated with different levels of of SRB.

Breads	Moisture	Protein	Fat	CH	Ash	PA	IDF	SDF	β-Glucans
Control	32.49 ± 0.44 ^b^	9.05 ± 0.03 ^b^	6.64 ± 0.39 ^a^	49.06 ± 0.24 ^e^	1.89 ± 0.06 ^a^	0.18 ± 0.02 ^a^	0.94 ± 0.12 ^a^	1.74 ± 0.12 ^d^	1.69 ± 0.12 ^bc^
SRB10	31.21 ± 0.26 ^a^	8.76 ± 0.35 ^ab^	8.37 ± 0.09 ^b^	48.54 ± 0.12 ^d^	2.44 ± 0.03 ^b^	0.52 ± 0.03 ^b^	4.32 ± 0.46 ^b^	1.43 ± 0.05 ^c^	1.70 ± 0.06 ^c^
SRB15	33.94 ± 0.05 ^c^	8.75 ± 0.07 ^a^	8.42 ± 0.09 ^b^	45.49 ± 0.14 ^c^	2.52 ± 0.08 ^b^	0.86 ± 0.01 ^c^	6.83 ± 0.16 ^c^	1.18 ± 0.04 ^b^	1.59 ± 0.05 ^ab^
SRB20	33.73 ± 0.15 ^c^	8.87 ± 0.03 ^ab^	9.12 ± 0.04 ^c^	44.48 ± 0.05 ^b^	2.80 ± 0.07 ^c^	1.10 ± 0.01 ^d^	12.78 ± 0.44 ^d^	0.99 ± 0.05 ^a^	1.59 ± 0.10 ^ab^
SRB25	33.90 ± 0.02 ^c^	8.96 ± 0.03 ^ab^	9.78 ± 0.13 ^d^	43.27 ± 0.17 ^a^	3.06 ± 0.07 ^d^	1.22 ± 0.02 ^e^	15.88 ± 0.47 ^e^	0.91 ± 0.05 ^a^	1.50 ± 0.05 ^a^

Data are the mean ± standard deviation of three independent experiments (n = 3). Different superscript letters indicate significant difference among mean values within a column (*p* ≤ 0.05 according to Duncan´s test). Abbreviations: CH, carbohydrates; IDF, insoluble dietary fiber; SDF, soluble dietary fiber; PA, phytic acid; SRB, stabilized rice bran; 10SRB, formulation with 10% of SRB; 15SRB, formulation with 15% of SRB; 20SRB, formulation with 20% of SRB; 25SRB, formulation with 25% of SRB.

## Data Availability

Not applicable.
